# Serological Protection 5–6 Years Post Vaccination Against Yellow Fever in African Infants Vaccinated in Routine Programmes

**DOI:** 10.3389/fimmu.2020.577751

**Published:** 2020-10-08

**Authors:** Olubukola T. Idoko, Cristina Domingo, Milagritos D. Tapia, Samba O. Sow, Christof Geldmacher, Elmar Saathoff, Beate Kampmann

**Affiliations:** ^1^Vaccines and Immunity Theme, Medical Research Council Unit The Gambia at the London School of Hygiene and Tropical Medicine, Fajara, Gambia; ^2^CIH^LMU^ Center for International Health, Medical Center of the University of Munich (Ludwig-Maximilians-Universität München), Munich, Germany; ^3^Centre for Biological Threats and Special Pathogens, Robert Koch Institute, Berlin, Germany; ^4^Centre pour le Développement des Vaccins, University of Maryland, Bamako, Mali; ^5^Division of Infectious Diseases and Tropical Medicine, University Hospital, Ludwig-Maximilians-Universität München Munich, Munich, Germany; ^6^German Centre for Infection Research (Deutsches Zentrum für Infektionsforschung), Munich, Germany; ^7^The Vaccine Centre, Faculty of Infectious and Tropical Diseases, London School of Hygiene and Tropical Medicine, London, United Kingdom

**Keywords:** serologic, protection, 5-6 years post vaccination, yellow fever, routine immunizations

## Abstract

**Introduction:** Although effective live attenuated yellow fever (YF) vaccines have been available for over 9 decades sporadic outbreaks continue to occur in endemic regions. These may be linked to several factors including epidemiological factors such as vector and intermediate host distribution or vaccine coverage and efficacy. The World Health Organization's research priorities include gathering systematic evidence around the potential need for booster vaccination with YF vaccine whether this follows full or fractional doses in children. Knowledge on the longevity of response to YF vaccine and the implications of this response needs to be consolidated to guide future vaccination policy.

**Methods:** We measured anti-YF IgG by microneutralization assay in a group of 481 African infants who had received YF vaccine as part of routine EPI programmes, to explore serological protection from YF 5–6 years post YF vaccination, as well as the effect of co variates.

**Findings:** Notably, 22.2% of the cohort had undetectable antibody concentrations, with another 7.5% revealing concentrations below the threshold of seropositivity of 0.5 IU/mL. Sex, season, country and time since vaccination did not affect the longevity of antibody concentration or having antibody concentrations above a defined threshold.

**Conclusion:** Roughly 30% of children in this cohort did not demonstrate anti-yellow fever antibody concentrations above the defined threshold of protection, with 20% having no demonstrable antibody. Knowledge on the longevity of response to YF vaccine and the implications needs to be consolidated to guide future vaccination policy.

## Background

Effective vaccines against yellow fever (YF) virus have been available for over nine decades (1). In endemic regions of the world, these vaccines are generally used as part of the routine Expanded Programme on Immunization (EPI) vaccines given to children in infancy (2). The currently available YF vaccines are live attenuated vaccines shown to be highly immunogenic and to provide long term protection after a single dose (1).The primary correlate of protection is neutralizing antibody, though cell mediated and innate immune responses have also been proposed to play a role (3).

In 2014 the World Health Organization (WHO) changed its recommendation of 10-yearly vaccination against YF to a single dose for life. Fractional doses are also in use during epidemics when vaccine supplies are limited. However, WHO has recognized the need for studies that establish the longevity of response to a single YF vaccine dose as a priority, particularly in special groups such as infants, immunocompromised individuals and those who received fractional doses of the vaccine (4–6). Establishing the longevity of response following single dose YF vaccination is key to guide future policy on the use of the vaccine, particularly in endemic settings. If the longevity of response is found to be sub optimal in these groups, millions may be vulnerable to infection in these endemic areas. The current projections of population coverage under the 2017–2026 Eliminating YF Epidemics (EYE) strategy, which implements YF vaccination in infants as part of the EPI in endemic countries (7), could be off-target if serological and possibly clinical protection from disease is short-lived.

The proportion of individuals with protective anti-yellow fever antibody reported in the literature ranges from 69 to 98% up to 11 years post vaccination (8–12). Data from children remains limited but are of particular importance, given that in endemic settings a single dose for life given in infancy would be the only YF vaccine administration. It is also possible that immune responses to YF differ between adults and children and data from children showing both production and longevity of protective antibody need to be generated, especially since it has already been demonstrated that infants show poorer and sometimes varied seroconversion rates following YF vaccination (13–16) in different settings. This finding is likely to impact on the longevity of antibody response.

To our knowledge there are limited data on longevity of YF antibody available from the African continent. One previous study by Domingo et al. involving children from Ghana and Mali examined longevity of antibody response to YF vaccine in African infants who had received the vaccines according to EPI schedules but as part of large randomized controlled trials (ideal settings). In these circumstances, “real life” factors such as cold chain maintenance and other programmatic limitations are less likely to affect overall outcome given the influence of full time study teams (17). Generating data on the antibody concentrations to YF several years post vaccination under routine EPI program conditions therefore remains important to inform the WHO guidelines with a view to assess need for booster doses if the only dose of YF vaccine is administered in infancy.

We measured anti-YF IgG by microneutralization assay in a group of African infants who had received YF vaccine as part of routine EPI programmes, to explore serological protection from YF 5–6 years post YF vaccination.

We also explored the impact of cofactors such as sex, season of vaccination, country and time since vaccination on the concentration of anti-YF IgG neutralizing antibodies.

## Role of Funding Source

The funders had no role in the study design, data collection, data analysis, data interpretation, or manuscript write up.

## Ethical Approvals

Written informed consent according Good Clinical Practice guidelines and the Declaration of Helsinki was obtained from a parent of each participant. The initial and current studies were approved by the ethics committees at the host institutions and Programme for Appropriate Technology in Health (PATH).

## Methods

### Population and Samples

We assayed YF antibody responses in a cohort of African children from The Gambia (*N* = 243) and Mali (*N* = 238) using banked serum samples which were originally collected as part of an antibody persistence study to assess persistence of antibodies to MenAfriVac 4–5 years after the children had originally received MenAfriVac at 12–23 months of age. With these 481 samples we would have a power of 1.0 to detect a 10% difference in the proportion of individuals attaining protective titres assuming that one vaccine dose would result in 98% of children having protective anti-YF antibody titres 5–6 years post vaccination at a 5% alpha.

These blood samples were collected between October 2011 and April 2012 and included samples from all children who could be traced 4–5 years post MenAfriVac vaccination. Left over banked serum was accessed for this analysis (18) and current assays were conducted between January and March 2019.

### Records of YF Vaccination

As a prerequisite to enrolment into the MenAfrivac trial at age 12–23 months, the infants were required to have documentation showing that they received all recommended vaccinations for age which included one dose of YF vaccine received up to 1-year pre enrolment. The EPI program in Mali utilized yellow fever vaccines from Institute Pasteur, SANOFI Pasteur and BIOMANGUINHOS. The Gambia EPI utilized vaccines from vaccines from Institute Pasteur only. All blood samples assayed in this study were collected between 5- and 6-years post YF vaccination (corresponding to 4–5 years post study enrolment) and remaining aliquots had been stored at −70°C at the University of Siena Sera Bank in Italy. Samples were maintained at this temperature while stored at and during transport form the clinical sites and during transport to the assay lab.

### Lab Assays

The concentrations of neutralizing antibodies to YF virus were tested at the Robert Koch Institute in Berlin using a microneutralization assay (17). Briefly, 100 TCDI50 infectious doses of a YF virus 17-D (Stamaril, Sanofi Pasteur, Val de Reuil, France) were incubated with serial 2-fold dilutions of sera before inoculation into Vero cells cultured in 96-well plates. The cells were then microscopically examined for cytopathic effect 7 days later. Reference serum samples were run with each plate to minimize batch effects and ensure suitability of assay.

### Cut Offs

Although neutralizing titres of ≥1:5 or 1:10 (17, 19) are considered a surrogate of protection (seroprotection), difference in in assay methods may limit comparability of results from different laboratories. The titres were thus standardized by conversion into antibody concentrations in IU/mL using a WHO international standard (WHO International Standard, NISBC 99/616 reconstituted at 143 IU/ml) to allow for comparability with other available data. This was done by comparison with two positive controls for yellow fever neutralizing antibodies included with every assay. These controls were calibrated at 426.82 and 106.7 IU/ml, respectively. Based on earlier studies (20–22), we applied a concentration threshold of ≥0·5 IU/ml to discriminate seropositivity.

### Data Analysis

Where applicable, antibody concentrations were normalized by log 2 transformation. Uni- and multivariable mixed effects models were applied, adjusting for sex, season of vaccination and time since vaccination. Separate models were run for seropositivity (binary outcome) and raw post-vaccination antibody titres (continuous). Means, median, proportions and odds ratios were calculated along with their corresponding 95% confidence intervals. An alpha error level of 5% was used to judge significance. All analyses were performed using Stata version 14·2 (23).

## Results

Sera from 481 children (238 from Mali and 243 from The Gambia) were available of which 224 were male and 256 were female. Sex was missing for one participant.

### Participants Demonstrating Seropositivity 5–6 Years Post Vaccination

The median antibody concentration was 1.2 [interquartile range (0.4–2.4)]. Notably, 22.2% of the cohort had undetectable antibody concentrations, with another 7.5% revealing concentrations below the threshold of seropositivity of 0.5 IU/mL. Table 1 summarizes antibody concentrations by country.

**Table 1:** Anti-YF antibody concentrations (UI/mL).

	**Mean (95% CI)**	**Percentage with undetectable antibody (% 95% CI)**	**Percentage with antibody below protective threshold (% 95% CI)**
Overall (*N* = 481)	2.9 (2.3–3.5)	22.2 (18.6–26.2)	29.7 (25.7–34.0)
Mali (*N* =238)	3.4 (2.3–4.4)	25.6 (20.2–31.7)	31.5 (25.7–37.8)
Gambia (*N* = 243)	2.5 (1.9–3.0)	18.9 (14.2–24.4)	28.0 (22.4–34.1)

*95% CI, 95% confidence interval; IQR, interquartile range; YF Ab, YF antibody; N, number of observations; NB, Percentage with concentrations below the protective threshold includes individuals with undetectable concentrations*.

The antibody concentrations did not differ significantly by country (*p* = 0.13) with a trend to higher concentrations in the Malian cohort.

### Participants With Defined Protective Antibody Titer

The median antibody titer was 1:12.0 [interquartile range (1:4.0–1:22.0)]. Of the cohort, 21.8% had undetectable antibody titres at this time point, with another 5% revealing concentrations below the defined titer for protection of 1:5.

### Distribution of Antibody Concentrations

Antibody concentration distribution was non- significantly higher concentrations in Mali (*t* = 1.51; *p* = 0.13) and showing generally low antibody tires 5–6 years post vaccination following a single dose of YF vaccine (Supplementary Figure 1).

### Effect of Covariates on Antibody Concentration

Sex, season, country and time since vaccination did not affect the longevity of antibody concentration in uni- or multivariable regression models for antibody concentration as a continuous variable (Table 2).

**Table 2:** Association of covariates at time of vaccination with log2 anti-YF antibody concentrations.

		**Univariable (crude)**		**Multivariable (adjusted)**	
**Covariate**	**N**	**Coef. (95% CI)**	**p-value**	**Coef. (95% CI)**	**p-value**
**Sex**					
Male^*^	256	0.00 - -	–	0.00 - -	–
Female	224	0.02 (−0.15 to 0.20)	0.7767	0.03 (−0.15 to 0.20)	0.7590
**Season**					
Dry^*^	311	0.00 - -	–	1.00 - -	–
Rainy	170	0.02 (−0.16 to 0.20)	0.8479	0.02 (−0.16 to 0.20)	0.8239
**Country**					
Mali^*^	232	0.00 - -	–	0.00 - -	–
Gambia	179	−0.04 (−0.21 to 0.13)	0.6343	−0.04 (−0.21 to 0.13)	0.6270
Time since vaccination	–	0.07 (−0.19 to 0.34)	0.5798	0.08 (−0.18 to 0.34)	0.5656

*NB, Sex missing for 1 participant*.

* *Reference stratum. N, number of observations; Coef, coeficient; 95% CI, 95% confidence interval. Results of separate uni- and multi-variable linear regression. Multi-variable model adjusted for, sex and season of vaccination and time since vaccination*.

The odds of maintaining an antibody concentration above 0.5 UI/mL was also unaffected by the sex, season of vaccination, country or time since vaccination (Table 3).

**Table 3:** Association of covariates at time of vaccination with recording a threshold of anti-YF antibody concentrations of 0.5 or more.

			**Univariable (crude)**		**Multivariable (adjusted)**	
**Covariate**	**N**	**Mean**	**OR (95% CI)**	***p*-value**	**OR (95% CI)**	**p-value**
**Sex**						
Male^*^	256	2.8	1.00 - -	–	1.00 - -	–
Female	224	3.0	1.12 (076–1.66)	0.5632	1.12 (0.75–1.65)	0.5846
**Season**						
Dry^*^	311	3.0	1.00 - -	–	1.00 - -	–
Rainy	170	2.8	0.21 (0.66–1.51)	0.9992	1.02 (0.68–1.54)	0.9102
**Country**						
Mali^*^	232	3.4	1.00 - -	–	1.00 - -	–
Gambia	179	2.5	1.20 (0.81–1.77)	0.3708	1.20 (0.81–1.77)	0.3652
Time since	–	–	1.05 (0.57–1.91)	0.8853	1.06 (0.58–1.92)	0.8510
vaccination						

*NB, Sex missing for 1 participant*.

* *Reference stratum. N, number of observations; Coef, coeficient; 95% CI, 95% confidence interval. Results of separate uni- and multi-variable logistic regression. Multi-variable model adjusted for, sex and season of vaccination and time since vaccination*.

Similar findings were noted when the cut off for titres of ≥1:5 or 1:10 was applied.

## Discussion

The analysis of serum samples from 481 children vaccinated with one dose of YF vaccine in infancy showed that roughly 30% did not have protective antibody concentrations of 0.5 UI/ml (17) 5–6 years after vaccination. Most of this group had undetectable antibody concentrations (seronegative) and even where detectable, concentrations were below 0.1 IU/mL. This finding is concerning and may have significant implications for long term protection from YF in individuals who have received the YF vaccine as a single dose in the first year of life within EPI programs and in line with the current WHO recommendations.

By comparison, a small study in adults from the Netherlands who had received a fractional dose of YF vaccine demonstrated that 98% of the individuals vaccinated were still protected 10 years later (10), but the sample size of 40 individuals was small and represented only 48% of the original recipients of the vaccine (10). A more recent report followed up 349 Chinese adults who had received YF vaccine prior to deployment to Africa and reported negligible concentrations 11 years post vaccination (12). Several other studies have demonstrated similar decline in antibody in adults at varied time points post vaccination (9, 11, 24–26). The findings following a fractional dose of YF vaccine in The Netherlands differ from ours with a larger proportion retaining protective concentration but are in keeping with other reports of declining antibody from adults. All of these data are generated from studies in adults, unlike the pediatric data presented here. In addition, the different geographical settings (Europe, Asia vs. YF endemic Africa) have no or different prior exposure and other environmental differences which could also play a role in acquisition and persistence of antibody. Natural exposure differs between non-endemic and endemic settings and potentially also between age groups.

Following a full dose of YF vaccine in US adults all individuals who received multiple doses of YF vaccine had protective concentrations, irrespective of the time since vaccination. When only one dose had been received however, 94% had protective concentrations if that dose was <10 years from testing. When tested 10 or more years later however, only 82% had protective concentrations (11). These findings would suggest that even in adults, booster vaccinations may be warranted in certain populations. This view was published in a recent opinion piece (8).

Only 69% of children in Brazil maintained protective concentrations 10 years after receiving a full vaccine dose (8, 9). To our knowledge there has only been one study on the African continent assessing antibody longevity in children. This recent study assessed longevity in children who had received YF vaccine as part of 2 randomized controlled clinical trials. The study similarly demonstrated a drastic decline of YF immunity in children vaccinated as infants even within the ideal clinical trial settings with 50.4% (4.5 years post vaccination) and 43.1% (6 years post vaccination) retaining seropositivity in Mali and Ghana, respectively (17). These findings may be compounded by several factors including natural disease exposure but in keeping with ours suggest a population of children vulnerable to YF infection several years post vaccination irrespective of setting where vaccine was received.

Given the limited data available on persistence of antibody to YF in children and previous similar findings, our findings are timely and have the potential to inform future discussions regarding policy for YF vaccine use. The data from Brazil however does not state the vaccine used and this information may be an important consideration for the longevity of vaccine response. Our cohorts received similar vaccines although we do not have data per individual for vaccines received.

The sex, season of vaccination and time since vaccination did not impact the longevity of anti-YF antibody concentration 5–6 years post vaccination. This is similar to findings from a US cohort of adult travelers 3–4 years post vaccination with YF vaccine prior to travel to endemic regions. The only factors associated with higher antibody concentrations 3–4 years post vaccination were early onset of detection and higher antibody concentration 1-month post vaccination (27). These were not variables available for our analysis. In African studies sex was also not found to contribute to seropositivity (17).

## Limitations

The precise age at vaccination of our cohort (no date of birth records available) and anthropometric measures at time of vaccination were unknown and could have impact on the longevity of antibody. There may also be limitations within the EPI system ranging from cold chain maintenance to vaccine delivery methods that could have affected our results. Given the similarity of our findings with those from children vaccinated in ideal clinical trial settings however (17), this is unlikely.

## Conclusion

As identified by WHO there is a need to gather evidence around the potential need for booster vaccination with YF vaccine whether this follows full or fractional doses in children and adults (4, 5, 28). This knowledge needs to be consolidated to guide future vaccination policy. Our findings suggest that children in this region of sub-Saharan Africa may require booster doses due at least by school entry age. Additional studies that explore antibody functionality and not just quantity would also be warranted as the function and not just the quantity of antibody is likely to mediate protection.

## Data Availability Statement

All datasets generated for this study are included in the article/Supplementary Material.

## Ethics Statement

The initial and current studies involving human participants were reviewed and approved by Medical Research Council Unit The Gambia/Gambian Government Joint ethics Committee and Comité d'éthique de la Faculté de médecine de pharmacie et d‘odonto-stomatologie (FMPOS), Bamako, Mali. Written informed consent to participate in the the original study as well as consent for future use of banked samples was provided by the participants' legal guardian/parent.

## Author Contributions

OI conceived the study and performed the statistical analysis and drafted the manuscript. CD led the performance of the laboratory assays and critically reviewed the manuscript content. OI, BK, SS, and MT led recruitment of participants for the original studies and reviewed the manuscript. ES provided support for statistical analysis and critically reviewed manuscript content. CG and BK supported analysis and critically reviewed the manuscript. All authors contributed to the article and approved the submitted version.

## Conflict of Interest

The authors declare that the research was conducted in the absence of any commercial or financial relationships that could be construed as a potential conflict of interest.

## Supplementary Material

Supplementary Figure 1 Distribution if anti-YF antibody concentration by Country. Kernel density: non-parametric estimation of probability density function of anti-YF concentration. NB: *P*-value for difference in antibody concentration between countries = 0.1348.
