# Supplementary figures and images for: Serological Protection 5–6 Years Post Vaccination Against Yellow Fever in African Infants Vaccinated in Routine Programmes
Source: Front Immunol. 2020 Oct 8;11:577751. doi: 10.3389/fimmu.2020.577751 (PMC7578390; doi:10.3389/fimmu.2020.577751)

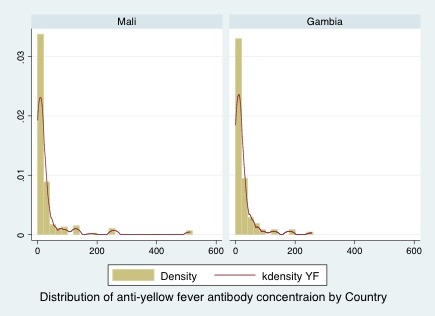

Supplement: Supplementary Figure 1 — Distribution if anti-YF antibody concentration by Country. Kernel density: non-parametric estimation of probability density function of anti-YF concentration. NB: P-value for difference in antibody concentration between countries = 0.1348. [file Image_1.jpeg]
